# Focal Cortical Dysplasia with hippocampal sclerosis

**DOI:** 10.4322/acr.2023.420

**Published:** 2023-01-23

**Authors:** Devendra Jadav, Vaibhav Gupta, Sudeep Khera, Vikas Meshram

**Affiliations:** 1 All India Institute of Medical Sciences, Department of Forensic Medicine and Toxicology, Jodhpur, Rajasthan, India; 2 Vardhman Mahavir Medical College and Safdarjung Hospital, Department of Forensic Medicine and Toxicology, New Delhi, India; 3 All India Institute of Medical Sciences, Department of Pathology and Lab Medicine, Jodhpur, Rajasthan, India

**Keywords:** Cortical Dysplasia- Focal Epilepsy Syndrome Histopathology, Immunohistochemistry, Forensic Pathology, Autopsy

## Abstract

Focal Cortical Dysplasia (FCD) is a group of focal developmental malformations of the cerebral cortex cytoarchitecture. FCD usually manifests as medically intractable epilepsy, especially in young children. Live patients are diagnosed by radiological examination such as magnetic resonance imaging (MRI), fluorodeoxyglucose positron emission tomography (FDG PET), magnetoencephalography (MEG), diffusion-tensor imaging (DTI), and intracranial electroencephalogram (EEG). While some cases can be missed by radiological examination, they are usually diagnosed on the histopathological examination of the surgically removed specimens of medically intractable epilepsy patients. We report a case of a young girl with cerebral palsy, mental retardation, and seizure disorder who died in her sleep. The deceased was diagnosed with FCD type III with hippocampal sclerosis on histopathological examination at autopsy. H & E stain and NeuN immunohistochemistry neuronal cell marker were used to demonstrate the findings of FCD.

## INTRODUCTION

FCD primarily refers to the focal developmental malformations in the cytoarchitecture of the cerebral cortex.^1^ These anomalies are developmental defects of the cerebral cortex, ultimately manifesting as epilepsy as well as severe neurological abnormalities, autism spectrum disorders, intellectual impairments, delayed development or worsening of cognitive aspects.^2^ The severity of FCD is determined by its site, general morphology, and microscopic features. The most trivial form of FCD is microdysgenesis, located in only a tiny portion of the cerebral cortex. However, it can be disseminated into an entire hemisphere, or both hemispheres, called a massive FCD.^2^ These lesions are usually associated with the hypoplasia of the affected region.^2^ Seizures in cases of FCD can start at any stage of life, predominantly from childhood.^3^ We present a case of FCD with severe neurological impairment and seizures in a young girl child in which diagnosis of FCD was established at autopsy.

## CASE REPORT

A 13-year-old female child was brought dead to an emergency. The body was shifted to the mortuary for post-mortem examination. As per the history, the deceased used to live in a Shelter home. She was a known case of cerebral palsy with mental retardation and seizure disorder. Previous clinical records were not available for evaluation, and information about her current medication could not be obtained. She was last seen alive at noon on the day she died. After having lunch, she fell asleep. However, she was found unresponsive in her sleep.

## AUTOPSY FINDINGS

**Figure 1:**
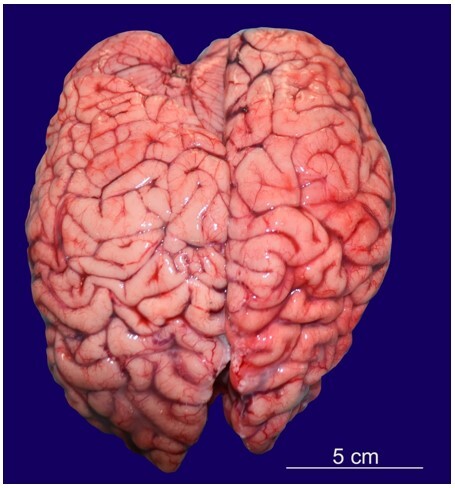
Asymmetrical cerebral hemispheres showing larger right frontal lobe and left occipital lobe.

On autopsy, muscle wasting was noted in both upper and lower limbs. Clubbing of nails was present. There was scoliosis of the spine with the convexity of the thoracic spine towards the right side and convexity of the lumbar spine towards the left side. The cranial cavity was asymmetrical, with the left side larger than the right side. The floor of the anterior cranial fossa was more arched. In the posterior cranial fossa, the left side was larger, deeper, and more spacious. In contrast, the right side was smaller and compressed. The brain weighed 632 g (Normal average weight of the brain at 13 years of age is 1243 g)^4^ and was grossly asymmetrical. The left occipital lobe was larger, whereas the right occipital lobe was smaller and compressed (Figure 1).

The right frontal lobe was larger than the left frontal lobe.

The right lung weighed 286 g and was larger than the left, which weighed 128 g. (Normal average weight for both lungs at 13 years of age is 602.3 g).^4^ Yellowish-green undigested food particles were present in the larynx, trachea, and main bronchi and were traced up to the terminal bronchi. The heart weighed 136 g (Normal average weight of a heart at 13 years of age is 207 g)^4^ and was unremarkable. A toxicological examination was negative for any drugs and toxins.

**Figure 2:**
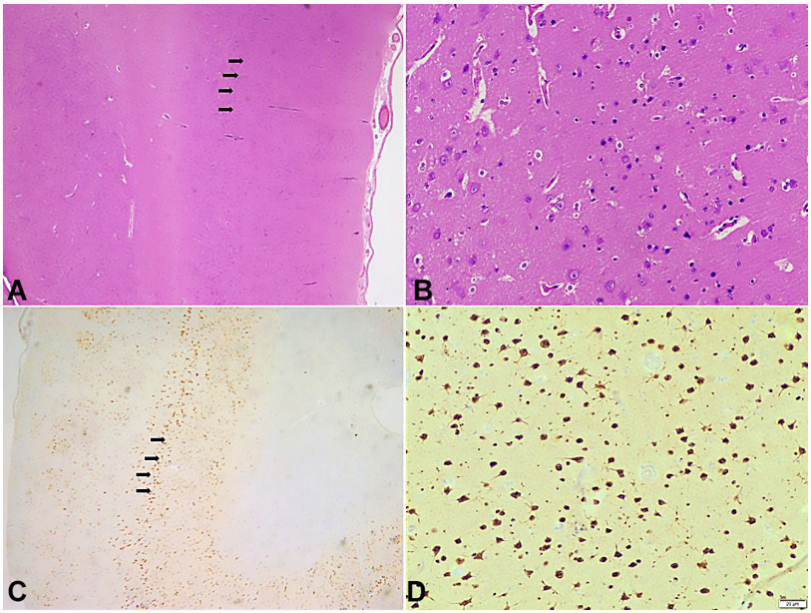
Photomicrography of the Central Nervous System. A - Tangential Disruption of cortical layers marked with black arrows (H&E, 100x); B - Higher magnification of tangential disruption of cortical layers without any identifiable dysmorphic neurons or balloon cells (H&E, 400x); C: NeuN highlights disordered arrangement of cortical layers marked with black arrows (100x); D: Higher magnification of cortical disruption of neurons highlighted by NeuN (400x).

Histopathological examination of multiple sections taken from different parts of the brain showed focal cortical dysplasia in the temporal lobe region in the form of tangential dyslamination in addition to areas of reactive gliosis (Figure 2).

**Figure 3:**
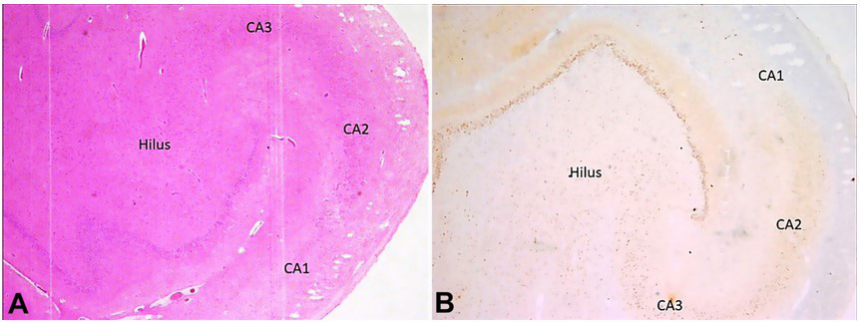
Photomicrography of the Central Nervous System - hippocampal region. A - Complete neuronal cell loss in CA1, marked depletion of neurons in CA3 and Hilus, and preserved neurons of the CA2 region of Hippocampus (H&E, 100x); B - NeuN highlights complete neuronal cell loss in CA1, marked depletion of neurons in CA3 and Hilus, and preserved neurons of the CA2 region of Hippocampus (100x).

There was attenuation of cortical layer III in the temporal lobe section. No dysmorphic cells/balloon cells were noted. Sections from the hippocampal region revealed a complete loss of CA1 neurons and marked depletion of CA3 and CA4 (Hilus) neurons, while neurons of the CA2 region were relatively preserved (Figure 3A and 3B). Sections taken from the cerebellum revealed a reduced number of Purkinje cells.

## DISCUSSION

FCDs are the most common cause of drug-resistant epilepsy in children.^3^ Although it is classified as a sporadic illness, the family incidence in some cases shows the involvement of various genetic pathways.^4^ The presentation of seizures depends upon the anatomical site of FCD.^1^ Sleep-related seizure marked by stereotyped bilateral movements and vocalizations, occasionally with retained awareness, can signify frontal lobe epilepsy. While patients with occipital lobe seizures may experience visual symptoms such as seeing colored dots or forms.^1^

FCDs have been classified variously based on histological changes since their conceptualization.^5^^-^ 7 Incipiently, FCDs have been classified into type I with altered cortical lamination and type II with neuronal cell dysmorphism. Palmini et al.^6^ further categorized them into subtypes. Type I was divided into Ia, Ib, and Ic based on alteration in cortical lamination, i.e., radial, tangential, or both.^6^ In our case, it was tangential dyslamination. Type II was divided into IIa and IIb based on the presence or absence of balloon cells and neuronal dysmorphism, respectively.^6^ In our case, no balloon cells/dysmorphic cells were present. The most recent classification of FCD was given by the International League Against Epilepsy (ILAE) in 2011.^7^ The ILAE categorization has the benefit of allowing pathological alterations contiguous to or associated with significant brain lesions classified separately as type III.

Based on the type of primary lesion associated with type I of FCD, type III has been divided into four subtypes: Type IIIa is linked to hippocampal sclerosis; Type IIIb is linked to low-grade developmental brain tumors; Type IIIc is linked to vascular malformations; Type IIId is related to any other major lesion acquired in childhood.^7^ On the contrary, if type IIa or IIb FCD is associated with any of the above-mentioned primary lesions, it should be considered a 'dual pathology' rather than type III FCD.^8^

The histological appearance of type III FCDs is similar to that of type I. It can be differentiated by the presence of an aberrant band of tiny and clustered “granular” neurons in the outer part of layer II of the cortex.^9^ Out of type III FCDs, type IIIa is the most common type encountered in clinical practice as well as from surgically resected samples.^10^ Our case belongs to type IIIa of the ILAE classification, as histological examination showed the presence of hippocampal sclerosis along with FCD. ILAE further divided type IIIa FCD into three types based on the predominance of neuronal cell loss and gliosis in various hippocampus regions.^7^ Type I refers to predominance in CA1 and CA4 regions, type 2 refers to predominance in the CA1 region, and type 3 refers to predominance in CA4 regions. This case belongs to type 2 with predominance cell loss in the CA1 region. In 2022, the International League Against Epilepsy (ILAE) proposed a new category in the classification of FCD, “no definite FCD on histopathology”. It should be used only when there is a clinical suspicion of FCD in a case of cortical epilepsy with an abnormality of a cortical organization that remains ambiguous and histopathological findings are not compatible with FCD I, FCD II, or FCD III; or when there is incomplete surgical removal or sampling of the tissue.^11^

At autopsy, FCD lesions are identified grossly as an area of cortical thickening with poor differentiation from the underlying white matter.^12^ The frontal lobe and the precentral gyrus are the most common locations.^12^ In live patients of focal refractory epilepsy, FCD is diagnosed with an MRI scan. In such cases, surgical resection may be possible.^10^^,^ 13 The surgical outcome depends on the type of FCD in such cases. However, research showed that out of 266 cases of surgically resected samples of refractory epilepsy, MRI-negative FCD was detected in 36 (13.5%) cases.^10^ Apart from MRI, other newer diagnostic modalities such as FDG PET, MEG, DTI, and intra-cranial EEG are being used in the diagnosis and management of FCD.^10^^,^ 13

Hippocampal sclerosis (HS) is the most prevalent lesional finding observed in individuals with temporal lobe epilepsy.^12^ It can be isolated HS or associated with the FCD IIIa. The association between HS and FCD IIIa ranges from 25-70%.^14^ Premature, recurrent, and persistent seizures originating in the hippocampus may interfere with the development of the neocortical temporal lobe, leading to the development of FCD.^4^ It has also been found in people with a sudden unexpected death in epilepsy.^12^ It can be unilateral or bilateral. Grossly, it appears as atrophy of the hippocampal region, which felt harder on the cut section.^12^ It may be accompanied by the dilatation of the temporal horn of the lateral ventricle and atrophy of the surrounding regions. Apart from routine H&E staining, Nissl staining, immunohistochemistry of neuronal cell markers such as NeuN, and biomarkers for astroglial injury are useful in diagnosing HS.^12^ In our case, apart from H&E, NeuN immunohistochemistry was used to diagnose FCD. The typical finding of type III FCD on histology is the neuronal cell loss in the hilar and CA1 region, called as end folium pattern of sclerosis, which was evident in this case.^15^ The neurons of the CA2 region remain preserved in such cases.^15^

The deceased was a known case of cerebral palsy with mental retardation and seizure disorder. Details about her medications and the imaging report were unavailable. Histopathology of the brain clinched the diagnosis of Focal Cortical Dysplasia with hippocampal sclerosis. Imaging modalities like MRI are helpful in the diagnosis of this condition; however, the literature shows that MRI scans may be negative in about 13% of cases, particularly in a milder form. Histopathological examination can be the gold standard for diagnosing FCD from surgically resected or autopsy specimens. Histopathology can be useful for studying FCD in more detail and can be paramount for further research on this condition.

## References

[1] Crino PB (2015). Focal cortical dysplasia. Semin Neurol.

[2] Pascual-Castroviejo I, Hernández-Moneo JL, Gutiérrez-Molina ML (2012). Focal cortical dysplasia. Clinical-radiological-pathological associations. Neurologia.

[3] Shaker T, Bernier A, Carmant L (2016). Focal cortical dysplasia in childhood epilepsy. Semin Pediatr Neurol.

[4] Fabera P, Krijtova H, Tomasek M (2015). Familial temporal lobe epilepsy due to focal cortical dysplasia type IIIa. Seizure.

[5] Mischel PS, Nguyen LP, Vinters HV (1995). Cerebral cortical dysplasia associated with pediatric epilepsy. Review of neuropathologic features and proposal for a grading system. J Neuropathol Exp Neurol.

[6] Palmini A, Najm I, Avanzini G (2004). Terminology and classification of the cortical dysplasias. Neurology.

[7] Blümcke I, Thom M, Aronica E (2011). The clinicopathologic spectrum of focal cortical dysplasias: a consensus classification proposed by an ad hoc Task Force of the ILAE Diagnostic Methods Commission. Epilepsia.

[8] Marucci G, Farnedi A, Giulioni M (2012). Reelin: a possible link between hippocampal sclerosis and cortical dyslamination in the setting of FCD type IIIa. Neurol Sci.

[9] Blumcke I, Spreafico R, Haaker G (2017). Histopathological findings in brain tissue obtained during epilepsy surgery. N Engl J Med.

[10] Wang X, Deng D, Zhou C (2021). Focal cortical dysplasia type iii related medically refractory epilepsy: mri findings and potential predictors of surgery outcome. Diagnostics (Basel).

[11] Najm I, Lal D, Alonso Vanegas M (2022). The ILAE consensus classification of focal cortical dysplasia: an update proposed by an ad hoc task force of the ILAE diagnostic methods commission. Epilepsia.

[12] Thom M (2007). The autopsy in sudden unexpected adult death: epilepsy. Curr Diagn Pathol.

[13] Kabat J, Król P (2012). Focal cortical dysplasia: review. Pol J Radiol.

[14] Giulioni M, Vornetti G, Marucci G (2018). Letter to the Editor. Focal cortical dysplasia type IIIa and isolated hippocampal sclerosis. J Neurosurg.

[15] Thom M, Zhou J, Martinian L, Sisodiya S (2005). Quantitative post-mortem study of the hippocampus in chronic epilepsy: seizures do not inevitably cause neuronal loss. Brain.

